# Predictors of Compensatory Sweating and Satisfaction Following Endoscopic Thoracic Sympathetic Chain Clipping for Palmar/Axillary Hyperhidrosis

**DOI:** 10.3390/jcm14020326

**Published:** 2025-01-08

**Authors:** Dania Nachira, Maria Letizia Vita, Antonio Giulio Napolitano, Adriana Nocera, Maria Teresa Congedo, Giovanni Punzo, Leonardo Petracca Ciavarella, Elisa Meacci, Stefano Margaritora

**Affiliations:** 1 Department of Thoracic Surgery, Fondazione Policlinico Universitario A. Gemelli-IRCCS, Università Cattolica del Sacro Cuore, 00168 Rome, Italy; marialetizia.vita@policlinicogemelli.it (M.L.V.); antoniogiulionapolitano@gmail.com (A.G.N.); mariateresa.congedo@policlinicogemelli.it (M.T.C.); leonardo.petraccaciavarella@policlinicogemelli.it (L.P.C.); elisa.meacci@policlinicogemelli.it (E.M.); stefano.margaritora@policlinicogemelli.it (S.M.); 2 Department of Anesthesiology and Intensive Care Medicine, Fondazione Policlinico Universitario A. Gemelli-IRCCS, Università Cattolica del Sacro Cuore, 00168 Rome, Italy; giovanni.punzo@policlinicogemelli.it

**Keywords:** primary hyperhidrosis, endoscopic thoracic sympathectomy, clipping, compensatory sweating, plantar hyperhidrosis, quality of life

## Abstract

**Background**: Endoscopic thoracic sympathetic chain clipping (ETSC) is a definitive treatment for primary palmar and/or axillary hyperhidrosis (PPAH); however, compensatory sweating (CS) remains a feared complication. The aims of this study were to investigate the factors associated with CS and satisfaction with the treatment and to evaluate the post-operative quality of life (QoL). **Methods**: From January 2011 to August 2023, 180 patients who had undergone two-stage ETSC were prospectively asked to complete pre- and post-operative questionnaires on satisfaction, CS, and QoL in several daily activities. **Results**: Seventy-nine patients (45.7%) were male, and fifty-two (30.1%) were active smokers, with a mean body max index (BMI) of 22.6 ± 3.14. The majority of the population (112 (62.2%)) was operated on for combined palmar and axillary primary hyperhidrosis (PH), whereas 56 (31.1%) patients had only palmar and 12 (6.7%) only axillar PH. Only 122 (67.8%) patients completed ETSC on both sides and the follow-up in the study period. CS was 50.8% (62 patients), and there was severe CS in 7 cases (5.7%); 9 (7.4%) patients developed a gustatory CS. The final effectiveness of ETSC was 95.9%, with a reported improvement in QoL in 95.3% of cases (mainly in manual work and socialization); 94.1% of patients were satisfied and would undertake ETSC again. At multivariable analysis, only older age (>24 years) was a predictor of CS (*p* = 0.007) and severe CS (*p* = 0.042). No predictor for satisfaction was found. **Conclusions**: ETS by clipping can improve QoL in cases of palmar/axillary hyperhidrosis. Older patients must be informed of a higher risk of CS.

## 1. Introduction

Primary palmar and/or axillary hyperhidrosis (PPAH) is a benign disorder of the sympathetic system that affects 2% of the population, causing excessive sweating in the affected areas and a consequent quality of life (QoL) impairment in patients’ daily-life activities [1].

Nowadays, endoscopic thoracic sympathetic chain clipping (ETSC) by Video-assisted thoracic surgery (VATS) is considered the definitive treatment for PPAH [2]; however, the development of compensatory sweating (CS) still remains unsolved and is the most feared adverse event (with an incidence of 50–80%, [3,4,5,6,7]), which can also compromise patient’s QoL in severe forms.

Several studies [3,4,5,6,7] have tried to evaluate the main predictors of CS after ETSC, and the most frequently proposed factors were older age, primary indication other than palmar hyperhidrosis, current smoking [6], body max index (BMI) > 25 to 28.5 [3,6], no plantar PH (primary hyperhidrosis) [6], and bilateral ETSC performed at the same time [3,4,5].

Deep learning of the risk factors potentially associated with the development of CS and predictors for the patient’s satisfaction can have a pivotal role in personalizing the surgical counseling before ETSC, ensuring that the surgeon is clearly able to illustrate the benefits and potential disadvantages related to the surgery according to patient’s specific characteristics.

The aims of this study were to investigate the main predictors of CS, severe CS, and satisfaction and to evaluate the post-operative quality of life (QoL) of patients who had undergone ETSC.

## 2. Material and Methods

### 2.1. Ethical Statement

This study was approved by the Ethical Committee (Università Cattolica del Sacro Cuore) (Prot.ID no. 5037/14) and was therefore conducted in accordance with the ethical standards of the Declaration of Helsinki and its later amendments. All patients provided informed consent to participate in the study, ensuring their clinical data were treated anonymously.

### 2.2. Study Design

The study was a prospective, single-center observational study (case series).

From September 2014 to August 2023, the clinical data of 180 patients who had undergone two-stage ETSC were prospectively recorded and analyzed. Strengthening the Reporting of Observational Studies (STROBE) checklist was used to report the results of the present study.

Patients were asked to complete a pre-operative and post-operative standardized questionnaire (2 weeks after each side surgery and on follow-up of at least 25 months) on satisfaction, CS, and QoL in several daily activities. The questionnaire used at our center was an implementation of the data collection sheet formulated by De Campos et al. [2,8]. Through this questionnaire, it was possible to collect precise information about the preoperative situation of patients in terms of QoL and level of discomfort in daily activities due to PH and then to re-evaluate their satisfaction for the same queries at two different time-point FUPs after surgery.

All patients who underwent ETSC had been affected by PPAH since childhood, with a BMI < 28 and no other causes of sweating (endocrine disorders, menopause, hyperthyroidism, lymphoproliferative diseases, etc., which were considered exclusion criteria). A mean heart rate at rest inferior to 55 bpm was considered a contraindication for surgery.

Indeed, during pre-operative evaluation (with blood tests, electrocardiography (ECG), and chest X-ray), in case of bradycardia (heart rate frequency > 55 bpm), a 24 h Holter ECG was required. Only patients with a median heart rate > 55 bpm were considered for surgical treatment, as recommended by the STS expert Consensus Conference for the surgical treatment of hyperhidrosis [9].

### 2.3. Surgical Technique

At our center, the ETSC was performed in 2 stages, and usually, patients chose the dominant side to be treated first. The second side was operated on at least one month later. Surgery was performed with general anesthesia, single-lung ventilation adopting a near-zero fluid balance protocol [10], and with the patient on lateral decubitus.

**Figure 1:**
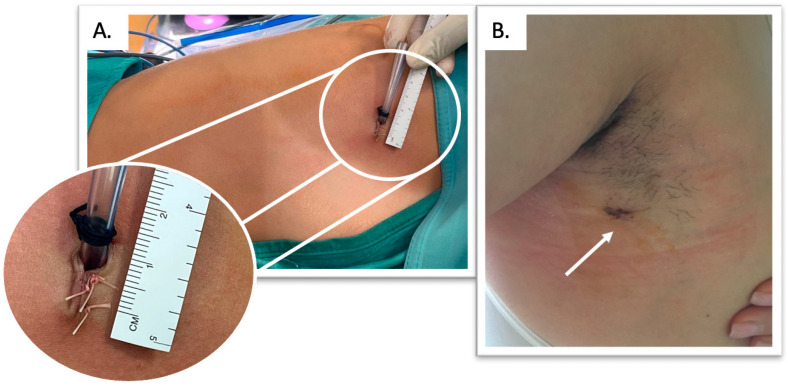
A 1 cm single-access endoscopic thoracic sympathetic clipping: (**A**). Surgical incision. (**B**). Surgical scar 7 days after surgery (white arrow).

From 2014 to June 2016 all procedures were performed by 5 mm biportal approach, then by 1 cm single-access approach with the incision performed in the III intercostal space on middle-axillary line [2], Figure 1. In all cases, a 5 mm 30° camera (Olympus^®^, Tokyo, Japan) was used, and the sympathetic chain was clipped by a 5 mm endoscopic titanium clip applier (EndoClip™, Medtronic^®^, Minneapolis, MN, USA) [2]. In the single-access approach, all the endoscopic instruments were inserted through the same incision, under the thoracoscope, one at a time. A 5 mm trocar (5 mm Bladless Optical Standard, Medtronic^®^, Minneapolis, MN, USA) was inserted in the upper part of the incision, to facilitate the introduction of the thoracoscope and prevent soiling, and then retracted to create enough space for the introduction of another instrument under the camera, through the same incision. A gently anti-Trendelenburg position and a 30–40° tilting of the patient on the ventral side were applied to improve the exposure of the sympathetic chain without the necessity of a further instrument for lung retraction.

**Figure 2:**
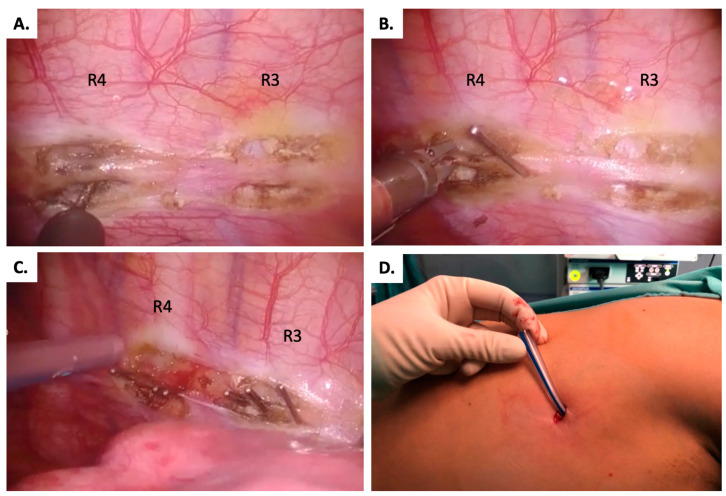
Intraoperative images of different steps of ETSC: (**A**). Dissection of the sympathetic chain by endoscopic electric hook at R3 and R4 levels. (**B**). Clip application at R4 top and bottom level by 5 mm endoscopic clip applier. (**C**). Final results of R3 top and bottom and R4 top and bottom ETSC, with chest tube inserted at the end of surgery. (**D**). Image of the surgical incision with chest tube inserted through the same space, before skin closure.

After the dissection of the sympathetic trunk, the clips were applied on the top and on the bottom of the ribs (according to the sweating area), using the rib-oriented nomenclature suggested in the expert consensus, Figure 2 [9]. For example, for patients affected by PPAH, R3 (top and bottom) and R4 (top and bottom) were clipped. In patients who complained of associated PH on the face, R2 bottom was also blocked, while for associated PH on the feet, R5 top was also clipped [9]. A careful exploration of the sympathetic chain was always performed to exclude the presence of Kuntz’s and Kirgis’ nerves, which must be cut, if present [2].

All patients underwent intercostal-nerve block by injecting 4 mL of 0.5% ropivacaine into the intercostal spaces (III–V) under thoracoscopic guidance [11] before closing the surgical incision, to reduce post-operative pain and discomfort. A 12–16 Fr chest tube was placed at the end of the operation and usually removed the day after surgery.

### 2.4. Primary and Secondary Outcomes

The primary outcomes of the study were to evaluate the incidence of CS, severe CS, and satisfaction. The secondary outcomes were to define the main predictors of CS, severe CS, and satisfaction. The level of improvement in quality of life (QoL) in daily life activities was also assessed.

### 2.5. Sample Size

The study was designed to evaluate the number of patients who developed a CS in a group of consecutive patients who had undergone ETSC at our center. Previous studies stated that the prevalence of CS is 80% [3,4,5,6,7]; therefore; given a power of 80% and a type I error of 5% (α), the sample size was estimated with the following formula for a descriptive research study:

$$Sample\;size\;(n)=\left[\left(Z_{1-\frac{\alpha }{2}}\right)\left(Z_{1-\frac{\alpha }{2}}\right)\left(p\right)\left(q\right)\right]/{\left(d\right)}^{2}$$

where *n* is the desired sample size, $Z_{1-\frac{\alpha }{2}}$ is the standardized value for the corresponding level of confidence (=1.28 for a power of 80% and type I error of 5%), *p* the expected prevalence based on previous researchers, *q =* 1 *− p*, and *d* the margin of error (5%). A 10% dispersion of patients at follow-up was also considered. Consequently, the estimated sample size was 116 patients.

### 2.6. Statistical Analysis

Continuous variables were expressed as mean ± SD or medians if not normally distributed, while categorical variables were as numbers and percentages. Categorical variables were compared by Chi-square test. Continuous variables were compared using the independent-sample Student’s *t*-test or the Mann–Whitney U-test if normally or non-normally distributed (according to the Shapiro–Wilk test). The ROC-curve model was used to assess the optimal cut-off for age and BMI associated with the risk of CS. Descriptive analysis was conducted on the whole population, while final inferential analysis was only conducted on patients who completed surgery on both sides during the study FUP and answered the post-operative questionnaire. Therefore, the analysis was performed evaluating specific and complete items, while patients with missing data for one of the selected outcomes were excluded. 

Univariable analysis was performed using the Cox regression model. Any variable with a *p*-value less than 0.20 at univariable analysis was included in a Cox proportional hazards regression model to investigate the adjusted effect of an independent variable (male sex, age > 24 years, BMI, smoking habits, anxiety, familiarity, type of hyperhidrosis, level of clipping) on CS, severe CS, and satisfaction.

A *p* < 0.05 was considered statistically significant. Statistical analysis was performed using IBM SPSS Statistics for Macintosh, Version 25.00 (Armonk, NY, USA).

## 3. Results

Among 180 patients, 79 (45.7%) were male, and 52 (30.1%) were active smokers, with a mean body max index (BMI) of 22.6 ± 3.14. The majority of the population (112 (62.2%)) was operated on for combined PPAH, whereas 56 (31.1%) patients had only palmar and 12 (6.7%) had only axillar PH.

The main clinical characteristics of the population are reported in Table 1.

The mean age of the first operation was 30.2 ± 11.5 (14–60) years.

Sixty patients (33.3%) underwent a 5 mm bi-portal approach, while 120 (77.7%) underwent 1 cm single-access VATS. The right side was chosen and operated first in 154 patients (85.6%).

The mean operative time was 50.2 ± 19.7 min, with no intraoperative complications. Mortality was null.

The mean chest tube length was 1.6 ± 1.1 days (median: 1 day). In 22 (12.2%) cases the chest tube length was longer (median: 2 days) due to residual pneumothorax at post-operative chest X-ray. Only two major complications (1.1%) were recorded: two cases of hematothorax, which required urgent re-operation due to bleeding from intercostal vessels. Twenty-three patients (12.7%) developed transitory paresthesia of the intercostal nerve, which resolved after 2–3 weeks spontaneously, whereas seven patients (0.4%) had a chronic neuralgia (more than 3 months) that required painkillers.

Forty-eight (26.7%) patients developed CS after first-side ETSC, mainly on the thorax (60.0%), contralateral hand (26.8%), and feet (13.2%), with a 100% effectiveness on the operated side. No difference was recorded in terms of CS between the right side operated on before the left side (*p*: 0.164).

Six out of the eight patients (75%) with Raynaud syndrome declared an improvement in symptoms related to the syndrome after ETSC. Among the 123 patients with associated plantar sweating, 12 (10%) declared a worsening of sweating, 19 (15.5%) an improvement, and the remaining others a stable situation.

Only 122 (67.8%) patients completed ETSC on both sides (median: 3 months between the two surgeries) and answered the follow-up questionnaire completely, in the study period. The mean follow-up was 30.2 ± 5.4 months.

CS after second-side ETSC was 40.2% (49 patients), with an effectiveness of 96.7%.

At final follow-up, CS was 50.8% (62 patients), with severe CS in 7 cases (5.7%); 9 (7.4%) patients developed a gustatory CS.

The main zones affected by CS were the thorax (51.6%), thighs (17.7%), abdomen (16.1%), and the feet (14.6%).

**Figure 3:**
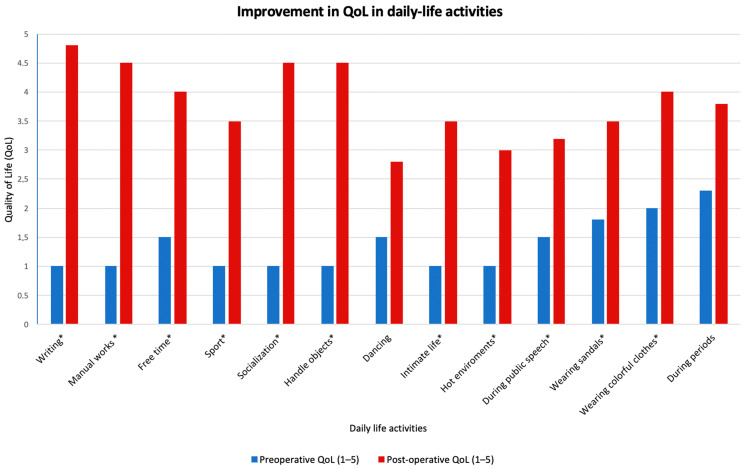
Histogram showing post-operative improvement in QoL during daily-life activities. * = statistically significant difference (*p* < 0.001).

The final effectiveness of ETSC was 95.9%, with a reported improvement in QoL in 95.3% of cases (mainly in manual work, writing, sport, socialization, and intimate life), see Figure 3. Aesthetic evaluation of surgical scar was optimal (98.2%).

A total of 94.1% of patients were satisfied with the results of the surgical treatment and would choose ETSC again. The less satisfied patients belonged to the group of patients with associated facial PH (*p* = 0.01), who also underwent R2 bottom clipping, due to post-operative CS symptoms and a higher recorded risk of developing gustatory sweating (7 out of the 9 patients who developed the problem, *p* = 0.035).

The variables associated with CS and severe CS at univariable analyses were smoking (*p* = 0.017), age > 24 years (*p* = 0.004), R2 bottom clipping (*p* = 0.038), and only age > 24 years (*p* = 0.032), respectively, as reported in Table 2 and Table 3.

At multivariable analysis, only older age (>24 years) was a predictor of CS (OR: 1.084, 95% CI [1.023–1.149], *p* = 0.007, Table 2) and severe CS (OR: 1.076, 95%CI [1.002–1.156], *p* = 0.042, Table 3).

No predictor for satisfaction was found.

## 4. Discussion

The results of our study provided significant insights into the outcomes in terms of QoL and risk factors associated with compensatory sweating (CS) following ETSC for palmar and axillary hyperhidrosis.

CS is a frequent and bothersome problem that affects patients after ETSC and that can compromise quality of life as much as PPAH itself. Therefore, it is extremely important for thoracic surgeons to be aware of the main factors associated with the development of CS in order to inform the patient about ETSC post-operative outcomes and customize surgery to balance the advantages and the risks according to the patient’s characteristics.

The incidence of CS is reported to be very high in some series, reaching 89% [12], with severe CS reaching 35–50.1% [12,13]. Recently, Alkosha [3] reported a 46% CS rate, with a 33% severe CS, in a series of 194 patients who underwent T3 or T3/T4 ETSC, for isolated palmar or palmo-axillary HH. The data were in line with our findings about the CS rate (50.8% in our series), but we recorded a lower incidence of severe CS (5.7% vs. 33% of the authors [3]), more in line with the 10.1% reported by Öncel et al. [14].

In our previous study [2], we recorded a higher overall CS of 56%, with a severe CS of 8%.

We can argue that the lower CS and severe CS in the present series, even lower than in our previous study [2], could be due to the relatively higher number of patients (68.3%) who had already presented with associated plantar PH (treated by R5 top clipping) at the time of surgery. These data suggest that the presence of pre-existing plantar sweating may act as a protective factor against the development of severe CS, in line with observations from Kargi et al. [15].

Indeed, to date, the main risk factors for CS reported in the literature are no plantar HH, BMI > 28.5, scalp HH, male sex, age over 24–31 years, multilevel clipping, T2 clipping, current smoking, and bilateral ETS performed at the same time [3,6,12,13,14,15,16,17,18], Table 4.

Interestingly, a unilateral dominant-side ETSC as the first step may lessen the risk of CS.

Two recent studies [4,5] also confirmed the efficacy of unilateral sequential ETSC in reducing the risk of CS.

We always used to perform ETSC in two consecutive stages, with the dominant side first, to reduce the patient’s post-operative pain and discomfort and the risk of longer hospitalization. Alkosha [5] confirmed our assumptions in a previous study, showing also how single-stage ETSC may improve plantar HH.

In our series, the only associated risk factor for the onset of CS at multivariable analysis was older age (over 24 years), which confirmed the findings of other studies [6,13,14,15,17,18].

An interesting and unexplained phenomenon recorded after ETSC, and linked to CS, is gustatory sweating exacerbated by the assumption of acid or spicy foods. In our series, gustatory sweating was quite limited, affecting only 7.4% of populations (mainly after R2 bottom clipping) vs. a higher rate in other series (38%, [12]).

Although the number of studies on ETSC outcomes is increasing, it is still difficult to compare the results among them, due to different techniques, surgical nomenclatures, and scales adopted to evaluate post-operative QoL, as already pointed out by the STS consensus conference [9]. For such reasons, the experts suggest that unified rib-oriented nomenclature and a standardized questionnaire are used (such as the one proposed by De Campos et al., [8]) for patient evaluation, suggestions that we adopted in our clinical practice.

Therefore, with limits related to the non-standardized management and outcomes evaluation in the literature, the current evidence suggests that clipping ganglia at higher levels, such as T2 or T3, can increase the risk of developing CS, especially in patients with only axillary sweating [19,20]. Some studies concluded that a more selective approach to ganglion clipping might reduce the risk of CS, thereby improving post-operative outcomes. For example, some authors [15,19] emphasized the importance of limiting clipping to relevant ganglia for specific treatment, thus avoiding an excessive extension of the intervention. The analysis of sympathectomy levels [19] showed that blocks at higher levels, such as R2, tend to eliminate most negative feedback signals, leading to a redistribution of sweating to other areas of the body, whereas blocks at lower levels preserve such signals, reducing the risk of CS in lower body areas. Adhami et al. [6] proposed a pathophysiological mechanism whereby the removal of sympathetic ganglia alters the balance of the autonomic nervous system, causing a redistribution of sweating to other areas of the body, which may explain the onset of CS in patients undergoing ETSC.

Regarding the extension of the sympathetic chain block, our approach frequently included clippings at R3, R4 (for PPAH), and R5 (for plantar-associated PH) levels, whereas some studies recommend limiting to T3 or T4 ganglia for hand and axilla sweating [20,21]. However, although a more selective clipping might reduce CS [20,21,22], our results indicated that the incidence of CS remained contained despite a more extensive clipping approach. Indeed, the R3–R4 block of the sympathetic chain can lead to less moist hands [22,23] without a significant increase in CS, above all if concomitant plantar hyperhidrosis is present [22].

Furthermore, at a longer mean FUP (30.2 ± 5.4 months), both CS and severe CS remained quite low in our series and were comparable to those recorded for only T3 (severe CS: 5%; [22,23]) or T4 (CS: 43.3%, severe CS: 6.7%, [20]) clipping vs. T3–T4 (CS: 87.5%; moderate–severe CS: 10–34.4%) of other studies [20,22].

Among 68.3% of patients with an associated plantar PH at the time of surgery, our results showed that R5 top clipping [9,23,24] provided an improvement in 15.5% of these patients, a stable situation in 75.5%, while a worsening of symptoms was observed in 10%.

Previously Neumayer et al. [25] showed that T4 clipping (top and bottom) can also provide satisfactory results on plantar sweating (experienced by 90.4% of the study population), with a reported improvement in 37.9% and a worsening in 15.2%. Compensatory sweating occurred in 19.4% of patients, rarely on the feet (only in 5.6% of cases).

Other authors [26] suggested blocking R5–R12 in addition to R4–R5 to obtain a significant improvement in plantar hyperhidrosis in patients with combined palmar and plantar PH.

Interestingly, Alkosha [5] showed that patients who had undergone two-stage bilateral R3 ETSC could have better improvement for plantar PH compared to single-stage surgery. In general, our experience supports the existing evidence in the literature that associated plantar PH to PPAH may act as a protective factor against the onset of CS, in particular on the feet, after ETSC. However, better treatment and the extension of the sympathetic chain block to obtain plantar sweating improvement have not yet been defined.

Concerning QoL post-surgery, it is known that it could decrease over time [22,23]; therefore, a long follow-up period is needed for a correct evaluation of ETSC outcomes. Our study revealed that CS increased from 40.2% (at 2 weeks FUP) to 50.8% at the final FUP, with a reduction in perceived effectiveness from 96.7% to 95.9%. The overall satisfaction was 94.1%, with a 95.3% reported improvement in QoL, particularly in daily activities and socialization, at a long FUP (mean: 32 months). However, patients who had undergone R2 bottom clipping (for associated facial PH) exhibited greater dissatisfaction in our series, underscoring the need for a careful evaluation of the risk profile for CS in this specific population.

In general, our QoL results are consistent with those of other authors [23,25,27], who highlighted a high degree of post-operative satisfaction (87.5–94%, [25,27], with improvement in QoL (95.9% at 30 days and 92.3% at 1-year FUP, [23]) despite the presence of CS (up to 97.6%).

In our opinion, the optimal post-operative results depend on the right selection of the patient for each procedure, and therefore pre-operative counseling still plays a pivotal role. Indeed, a careful evaluation of the patient, and of his potential risk for CS, needs, expectations, and involvement in the decision-making process by his surgeon, may help the patient to accept ESTC outcomes consciously and to improve his post-operative QoL.

### Limitations and Point of Strengths

This study had several limitations: the series was limited, with a monocentric setting, and not all patients completed bilateral surgery in the study period. As a case series, it lacked a control group to allow a comparative analysis of the outcomes. Furthermore, the population was heterogeneous, mainly in terms of age and types of PH, and also involved patients affected by plantar or facial PH. Unfortunately, the long-term QoL questionnaire was not administered at a precise time point to all patients, preventing us from having a precise idea of the possible CS increase and QoL decrease over time. Moreover, to better explore this aspect, an even longer FUP would be required.

However, the study also had important strengths. It was a prospective study, with good power, that provided a comprehensive analysis of risk factors, quality of life, and surgical outcomes associated with ETSC, with a long follow-up.

## 5. Conclusions

In conclusion, ETSC by clipping can improve QoL in cases of PPAH if a personalized approach is adopted. R3–R4 (top and bottom) ETSC seems to be a safe and effective treatment for PPAH. R5 top should be considered in patients with associated plantar PH. Older patients (age > 24 years) must be informed of a higher risk of CS; however, this does not seem to affect patients’ satisfaction and QoL improvement.

## Figures and Tables

**Table 1:** Clinical characteristics of the patients.

	**180 Patients (%)**
Sex (male)	79 (45.7%)
Mean age (years)	30.2 ± 11.5
Mean age HP onset (years)	9.8 ± 5.8
Smokers	52 (30.1%)
Anxiety	64 (35.5%)
Familiarity	46 (25.5%)
BMI	22.6 ± 3.14
Palmar sweating	56 (31.1%)
Axillary sweating	12 (6.7%)
Palmar + axillary sweating	112 (62.2%)
Facial sweating associated	23 (12.7%)
Plantar sweating associated	123 (68.3%)
Raynaud syndrome	8 (4.4%)
Previous nonsurgical treatments	65 (36.1%)

**Table 2:** Univariable and multivariable analyses for predictors of CS. Numbers in bold are significant (*p* < 0.05).

	**Univariable Analysis**	**Multivariable Analysis**	
	***p*** **-Value**	**OR [95% CI]**	***p*** **-Value**
Male sex	0.437		
Anxiety	0.438		
Familiarity	0.990		
Smoking	**0.017**		
Age ≥ 24 years	**0.004**	1.084 [1.023–1.149]	**0.007**
BMI ≤ 28	0.076		
R2 bottom clipping	**0.038**		
R5 top clipping	0.991		
Plantar HP	0.146		
Facial HP	0.123		

**Table 3:** Univariable and multivariable analyses for predictors of severe CS. Numbers in bold are significant (*p* < 0.05).

	**Univariable Analysis**	**Multivariable Analysis**	
	***p*** **-Value**	**OR [95% CI]**	***p*** **-Value**
Male sex	0.117		
Anxiety	0.408		
Familiarity	0.894		
Smoking	0.698		
Age ≥ 24 years	**0.032**	1.076 [1.002–1.156]	**0.042**
BMI ≤ 28	0.217		
R2 bottom clipping	0.245		
R5 top clipping	0.897		
Plantar HP	0.698		
Facial HP	0.346		

**Table 4:** Incidence of CS, severe CS, and risk factors according to the recent literature.

**Authors**	**Year**	**N**	**CS%**	**Severe CS%**	**Risk Factors for CS**
Licht and Pilegaard [12]	2004	131	89.0	35.0	Level T2-T4
De Campos et al. [8]	2005	102	95.1	24.7	T2–T3 and BMI
Miller et al. [17]	2009	282	/	21.0	Multilevel, age > 31 y, BMI > 28 kg/m^2^
Araújo et al. [13]	2009	80	85.0	32.4	BMI, male sex, extent of denervation, age
Öncel et al. [14]	2013	335	/	10.1	Older age, scalp-facial, T2
Bell et al. [18]	2014	191	75.0	19.0	Older age, scalp-facial, and axillary
Kargi et al. [15]	2016	95	42.1	12.6	BMI, age, level, and facial blushing
Adhami et al. [6]	2023	298	73.2	10.4–30.0	Older age, primary indication other than palmar hyperhidrosis, current smoking
Alkosha et al. [3]	2023	194	46.0	33.0	BMI < 28.5, absence of plantar HH, bilateral VATS
Present study	2024	180	50.8	5.7	Older age > 24 y

CS = compensatory sweating; BMI = body max index; HH = hyperhidrosis; VATS = Video-Assisted Thoracic Surgery.

## Data Availability

The data presented in this study are available on request from the corresponding author.
